# How are maternal and fetal outcomes incorporated when measuring benefits of interventions in pregnancy? Findings from a systematic review of cost-utility analyses

**DOI:** 10.1186/s12955-024-02293-4

**Published:** 2024-09-11

**Authors:** Lucy Abel, Helen Dakin, Ting Cai, Richard J. McManus, Abigail McNiven, Oliver Rivero-Arias

**Affiliations:** 1 Nuffield Department of Primary Care Health Sciences, University of Oxford, Oxford, UK; 2 Health Economics Research Centre, University of Oxford, Oxford, UK; 3 National Perinatal Epidemiology Unit, University of Oxford, Oxford, UK

**Keywords:** Pregnancy, Cost-utility analysis, Quality-adjusted life years, Maternal health

## Abstract

**Objective:**

Medical interventions used in pregnancy can affect the length and quality of life of both the pregnant person and fetus. The aim of this systematic review was to identify and describe the theoretical frameworks that underpin outcome measurement in cost-utility analyses of pregnancy interventions.

**Methods:**

Searches were conducted in the Paediatric Economic Database Evaluation (PEDE) database (up to 2017), as well as Medline, Embase and EconLit (2017–2019). We included all cost-utility analyses of any intervention given during pregnancy, published in English. We conducted a narrative synthesis of: study design; outcome construction (life expectancy, quality adjustment, discount rate); and whether the Incremental Cost-Effectiveness Ratio (ICER) was constructed using maternal or fetal outcomes. Where both outcomes were included, methods for combining them were extracted.

**Results:**

We identified 127 cost-utility analyses in pregnancy, of which 89 reported QALYs and 38 DALYs. Outcomes were considered solely for the fetus in 59 studies (47%), solely for the pregnant person in 13 studies (10%), and for both in 49 studies (39%). The choice to include or exclude one or both sets of outcomes was not consistent within particular clinical areas. Where outcomes for both mother and baby were included, methods for combining these outcomes varied. Twenty-nine studies summed QALYs/DALYs for maternal and fetal outcomes, with no adjustment. The remaining 20 took a variety of approaches designed to weigh maternal and fetal outcomes differently. These include (1) treating fetal outcomes as a component of maternal quality of life, rather than (or in addition to) an independent individual health outcome; (2) treating the maternal-fetal dyad as a single entity and applying a single utility value to each combination of outcomes; and (3) assigning a shorter time horizon to fetal outcomes to reduce the weight of lifetime fetal outcomes. Each approach made different assumptions about the relative value of maternal and fetal health outcomes, demonstrating a lack of consistency and the need for guidance.

**Conclusion:**

Methods for capturing QALY/DALY outcomes in cost-utility analysis in pregnancy vary widely. This lack of consistency indicates a need for new methods to support the valuation of maternal and fetal health outcomes.

**Supplementary Information:**

The online version contains supplementary material available at 10.1186/s12955-024-02293-4.

## Introduction

Pregnancy poses a unique challenge to methods of economic evaluation, and in particular cost-utility analysis (CUA). Interventions given in pregnancy have the potential to affect a range of health outcomes for both the pregnant person and fetus. In addition, those same health outcomes may be desirable or undesirable depending on the particular individual and context, for example in the case of unwanted pregnancy [1].

It is unclear how much weight (if any) should be given to fetal outcomes in cost-utility analysis. In both medical and research ethics, the valuation of fetal outcomes remains a topic of debate [2–4]. Estimating fetal quality-adjusted life year (QALY) gains in situations where termination of pregnancy or stillbirth are possible raise not only methodological challenges, but also difficult ethical questions about how to value potential future life years [5]. Consequently, studies frequently report cost-effectiveness of interventions in condition-specific terms, such as cost per outcome averted [6]. A decision-maker allocating resources must therefore decide individually what their willingness to pay is, per outcome, which can result in inconsistency between decision-makers, and across health conditions [7]. Thus decision-makers are left with inadequate tools to evaluate interventions for health conditions in pregnancy. This is a particular concern for health conditions that place the health of the pregnant person and fetus in tension, such as pre-eclampsia, where improving outcomes for the fetus, through prolonging gestation, increases the risk of maternal morbidity and mortality [8].

Previous work has found that the choice of outcomes reported in published economic evaluations in pregnancy and fertility tends to favour the intervention being evaluated [9], and that the decision to include or exclude fetal outcomes has the potential to affect cost-effectiveness estimates substantially [10]. The aim of this paper was to investigate the theoretical frameworks used for estimating and combining QALYs and disability-adjusted life years (DALYs) in pregnancy, by synthesising evidence from of cost-utility analyses within pregnancy using a systematic review. In particular, we answered the following three questions:

- How frequently are both maternal and fetal outcomes included in CUAs of pregnancy?
- How are fetal QALYs and DALYs constructed, particularly in cases such as fetal loss?
- Where both maternal and fetal outcomes are evaluated, how are these combined, and what assumptions are made in the process?

## Methods

The systematic review protocol was registered on PROSPERO [11]. The inclusion criteria were:

- CUAs – defined as studies reporting both costs and outcomes for an intervention and at least one comparator that measure outcomes in either QALYs or DALYs.
- Evaluations of interventions in pregnancy – defined as any intervention that was administered during pregnancy from conception to birth, including labour.
- Published in English.

The exclusion criteria were:

- Conference abstracts.
- Reviews or any paper that did not include a novel CUA.
- Interventions administered pre-conception or postpartum.
- Interventions applied to a broad population were included only if the costs and outcomes for the pregnant subgroup were reported separately.

Two search strategies were adopted concurrently and the results combined. For studies published before January 2017, the Pediatric Economic Database Evaluation (PEDE) database of paediatric economic evaluations was searched for terms relating to pregnancy [12]. At the point that searches were conducted, the PEDE database had not been updated since 2017. For studies published from January 2017 onwards, the PEDE search strategy [13] was adapted and replicated to identify pregnancy studies. Searches were conducted of Embase, Medline and EconLit in December 2019 and results were imported into Rayyan [14] for screening. Rayyan is online software for systematic reviewing that allows duplicate screeners to work on the same review.

Titles and abstracts were screened in duplicate (LA and TC). Economic evaluations were identified as “included”, and then labelled according to the type of analysis (cost-utility, other cost-effectiveness analysis, cost-benefit, cost-consequence, or unclear). Disagreements were resolved through discussion. We were able to resolve all disagreements through discussion, but would have consulted a third author had that been necessary.

Included articles were extracted using a pre-specified data extraction template (Appendix 1). This captured citation information, country, intervention type, outcome measure reported, whether outcomes reported were maternal or fetal, and how QALYs and DALYs were constructed. Construction refers to the set of assumptions made during the process of estimating QALYs and DALYs for a given health outcome. These include the methods used to capture quality of life effects, the duration that particular conditions are expected to affect quality of life, the life expectancy associated with different outcomes, and the discount rate.

Extracted data was synthesised narratively. We looked at how maternal and fetal outcomes were combined, and any frameworks or assumptions that were applied to balance fetal outcomes relative to maternal ones.

Quality assessment of papers was not performed as the aim of this review was to provide a description of the different methods used, rather than appraising the results using quality assessment.

## Results

**Fig. 1:**
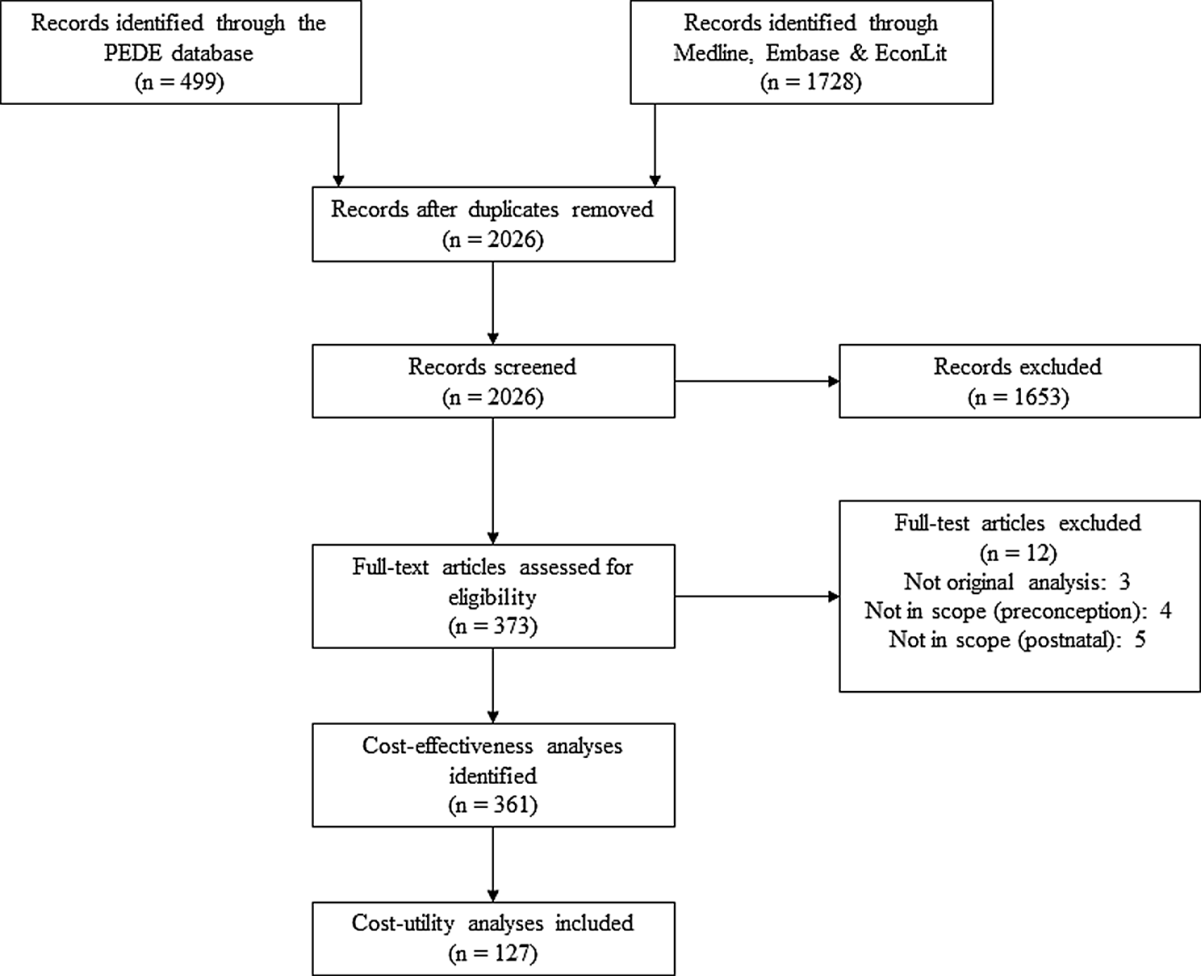
PRISMA diagram of systematic review

**Fig. 2:**
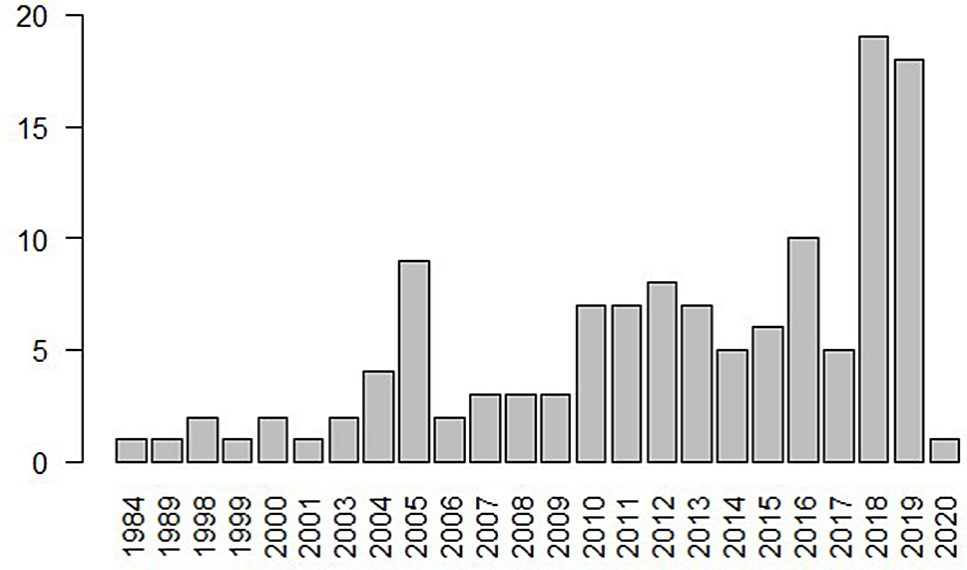
Number of studies published per year

Our search identified 127 CUAs reporting either QALYs (*n* = 89) or DALYs (*n* = 38) (Fig. 1). Publication dates ranged from 1984 onwards (Fig. 2).

Included studies covered a wide range of high, middle and low income countries, although most studies were either based in North America (*n* = 54, 43%) or Sub-Saharan Africa (*n* = 32, 25%). Studies covered a wide range of conditions, with HIV the most common condition (*n* = 21, 17%). In total, 50 conditions and 23 intervention types were analysed (Table 1).

Ninety-nine studies (87%) used a decision-analytical model in their analysis, 13 (10%) analysed individual participant data (such as within-trial analyses), and 11 (9%) used both methods. Four studies (3%) included neither models nor individual-patient data and instead synthesised pre-existing estimates of costs and effects across a set of comparators. For example, one study evaluated a range of interventions for reducing maternal and neonatal morbidity and mortality in low-income settings, using pre-existing estimates [15]. All models included were decision trees leading to outcomes that were extrapolated over the remaining life expectancy, or up to the time horizon of the study.

The vast majority of analyses took a lifetime horizon (*n* = 105; Table 1). Four followed fetal outcomes to adulthood (18 or 20 years), 10 did not clearly report the time horizon, and the remainder used a shorter horizon ranging from delivery to 10 years after the intervention. Three studies used separate time horizons for maternal and fetal outcomes.

All studies using a time horizon longer than a year applied discounting, with 3% being the most common value. No studies reported using different discount rates for maternal or fetal outcomes.

**Table 1:** Summary of characteristics of included studies

Characteristic	Number of studies (%)
**Region**	
East Asia & Pacific	12 (9)
Europe & Central Asia	29 (23)
Latin America & Caribbean	2 (2)
Middle East & North Africa	3 (2)
North America	54 (43)
South Asia	6 (5)
Sub-Saharan Africa	32 (25)
Unspecified	3 (2)
**Condition**	
Fetal abnormality	10 (8)
Fetal condition	1 (1)
Fetal Immunological condition	8 (6)
Infection	62 (49)
Labour complication	14 (11)
Maternal behaviour	3 (2)
Maternal condition	11 (9)
Maternal mental health	1 (1)
Maternal nutrition	3 (2)
Placental abnormality	2 (2)
Pre-existing maternal condition	2 (2)
Preterm birth	7 (6)
Various	5 (4)
**Intervention type**	
Antenatal care	1 (1)
Behavioural	3 (2)
Clinical decision rule	1 (1)
Delivery method	9 (7)
Delivery timing	1 (1)
Exercise program	1 (1)
Fetal monitoring	1 (1)
Mobile Health (mHealth)	1 (1)
Pharmaceutical	29 (23)
Policy	5 (4)
Screening	54 (43)
Supplementation	5 (4)
Surgery	2 (2)
Surrogacy	1 (1)
Training	2 (2)
Vaccination	11 (9)
Various	3 (2)
**Analysis type**	
Model	99 (78)
Trial	13 (10)
Both	11 (9)
Other	4 (3)
**Time horizon**	
Delivery	1 (0.8)
48 h post-delivery	1 (0.8)
3 months	2 (1.6)
1 year	2 (1.6)
2 years	1 (0.8)
10 years	1 (0.8)
18 years	1 (0.8)
20 years	3 (2)
Maternal lifetime	8 (6)
Fetal lifetime	97 (76)
Not reported	10 (8)

### Outcomes

Most studies reported fetal outcomes, either with maternal outcomes (49 studies, 39%) or without (59 studies, 46%). By contrast, only 13 studies (10%) reported solely maternal outcomes. Where outcomes are excluded, the analysis assumes either that those outcomes are unaffected by the intervention, or that no value is placed on any change in those outcomes. It was unclear which outcomes were included in one study [16]. In five studies, fetal outcomes were included, but it was unclear whether maternal outcomes were included.

**Fig. 3:**
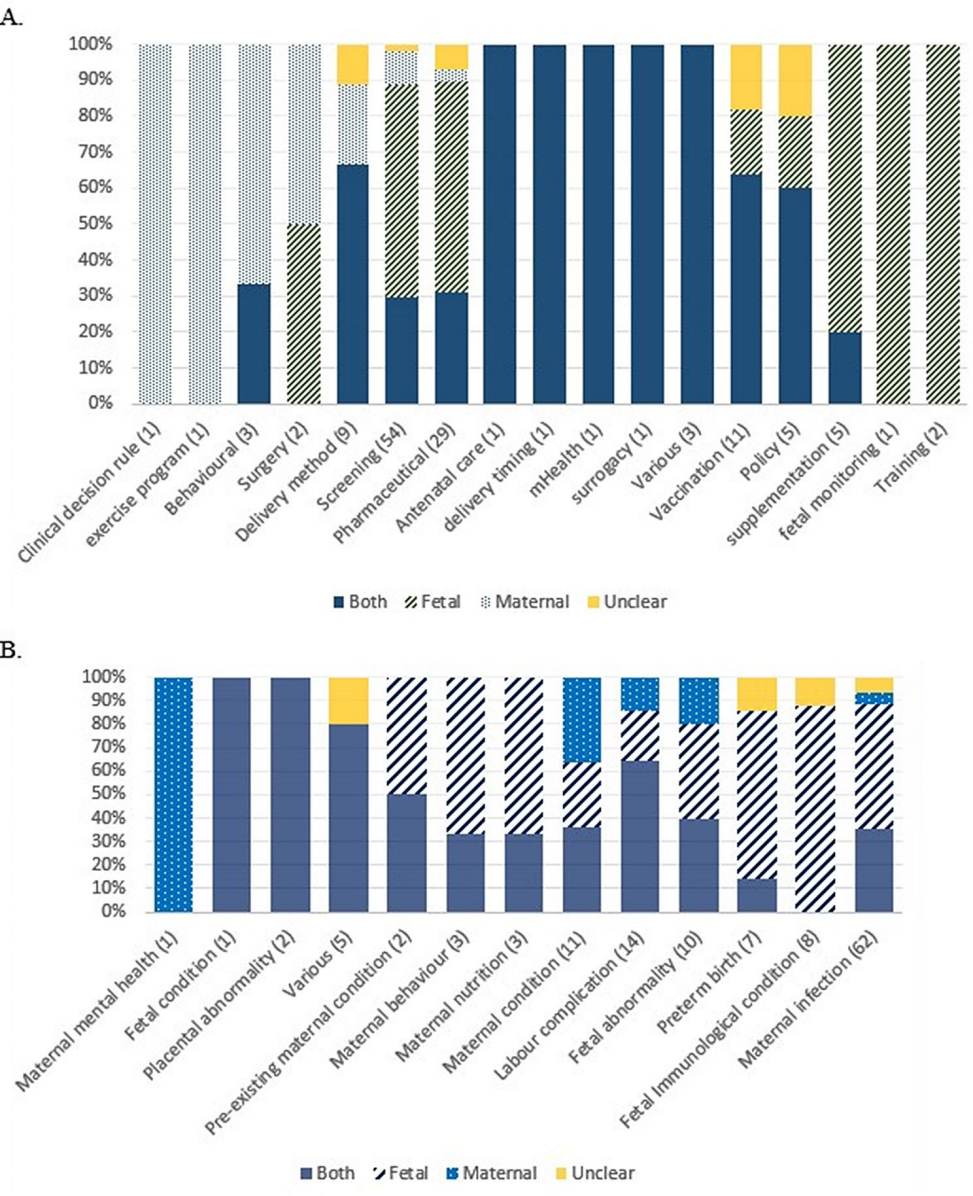
Percentage of studies reporting only maternal, only fetal or both outcomes, by (**A**) intervention type and (**B**) condition type. (N) Indicates number of studies

The inclusion or exclusion of maternal or fetal outcomes varied between and within intervention types and conditions (Fig. 3). Occasionally, there was also a discrepancy between who the authors stated the intervention was intended to benefit, and which outcomes were included. In three studies, the authors described the intervention as intending to benefit the fetus, but reported solely maternal outcomes in their evaluation. Twenty-three studies reported the intervention as intending to benefit the fetus primarily, but reported outcomes for both. For 42 studies the intervention was described as benefiting both pregnant person and fetus. Of these, 27 reported outcomes for both, five reported only fetal outcomes, seven only maternal, and, in the remaining three it was unclear.

Studies that took a lifetime horizon estimated QALYs or DALYs from clinical outcomes using an estimated life expectancy and one or more utility values. Typically, decision trees modelled the care pathway up to delivery, and then a health state was assigned to the pregnant person and infant at birth. This health state was assigned a utility and life expectancy. In some cases, the utility was assumed to change at some future point, for example when the child reached a certain age.

A common health outcome was a normal recovery, in which both pregnant person and baby were assumed to return to full health. A variety of methods were used to assign utilities to these outcomes, including population norms (9 studies), age-adjustment (5), and extrapolation (1). In 52 studies (41%), a utility of 1 was assigned for the entire lifetime where mother and baby were unaffected by negative health outcomes.

Fetal life expectancies often varied by outcome, to cover infants affected and unaffected by conditions such as cerebral palsy. The unaffected life expectancy was typically taken from national estimates such life tables, while the affected was drawn from relevant literature. Maternal life expectancies were generally calculated as the difference between the relevant national estimate and average maternal age.

Incomplete or unclear reporting was a common occurrence across studies. Of the 62 studies reporting maternal outcomes, 39 (63%) did not report the source of utility values. Of the 23 that did report utilities, sources included Global Burden of Disease adjustments (GBD – 5 studies), expert opinion (5), direct preference elicitation (4), values obtained from published literature (3), EQ-5D-3 L (3), SF-6D (2) and the Quality of Wellbeing Scale (1). One study used the SF-6D, but conducted sensitivity analysis using the EQ-5D-5 L [17]. Of the 108 analyses reporting fetal outcomes, 24 (22%) reported utilities. Sources included GBD (13), direct preference elicitation (4), published literature (3), HUI-3 (2), EQ-5D-3 L (1) and expert opinion (1). Life expectancies used to construct lifetime QALYs/DALYs were similarly underreported; 27 maternal values for life expectancy (44%) and 38 fetal values (34%) were missing.

### Methods for combining outcomes

In total, 53 studies included both maternal and fetal outcomes in their cost-utility analysis. A variety of methods were used to combine these outcomes for the purpose of the analysis (Table 2).

#### Additive

This was the simplest and also the most common method used by 31 studies (59%). QALYs or DALYs were summed across both pregnant person and baby with equal weight, and any discounting was applied at the same rate. The value judgement implied here is that all QALYs are equal, regardless of whether they accrue to pregnant person or fetus, and that changes in fetal health outcomes should be valued as they would be postnatally.

#### Dual time horizon

Three studies followed the additive approach, but used different time horizons for maternal and fetal outcomes, typically a shorter maternal time horizon followed by an extended fetal one. Two studies took a maternal time horizon up to delivery, and then a separate lifetime or 20 year horizon for fetal outcomes [18, 19]. Another study combined a fetal lifetime horizon with a 10 year maternal horizon [20].

The judgement implied here is that the intervention being analysed has differing effects on the pregnant person and fetus, and that these effects are of different durations. This might be the case if, for example, the condition is potentially fatal in the case of the fetus, but has only short-term health effects for the mother. In that case the shorter maternal time horizon would reflect that the outcomes beyond the time horizon will be identical regardless of which intervention they receive, rather than a judgement on the relative value of those outcomes.

Alternatively, in cases where the effects are known to continue beyond the time horizon (for example, a childhood time horizon where one outcome is a lifelong disability), the effect is to reduce the value of health outcomes for the individual for whom a shorter time horizon is used, relative to health outcomes that accrue for the other individual. In this review all three studies used a shorter time horizon for maternal outcomes. If maternal health effects from the intervention could plausibly persist beyond the time horizon of analysis, this would have the effect of devaluing maternal health outcomes relative to fetal health outcomes.

#### Maternal disutility

Five studies valued fetal outcomes as a maternal utility decrement. For example, *one* study valued maternal utility following labour and live birth as 0.99, and maternal utility following labour with a fetal death as 0.91 [21].

Three studies included a maternal disutility associated with fetal loss as part of an additive approach that included both maternal and fetal QALYs [22–24]. A fourth study expanded this to include both parents, who participated in a time trade-off to ascertain the parental disutility associated with having a child with cystic fibrosis [25].

The value judgement here is that fetal health outcomes are not valued as a separate individual, but that the mental health toll of fetal loss or disability is a component of maternal (or parental) health outcomes.

#### Dyad

One study treated the maternal-fetal dyad as an entity with a single outcome [26]. In this approach, life expectancies of mother and infant were added together, and then a utility value derived from expert opinion was assigned based on their combined health outcomes. The dyad was assigned 131 life years (76 fetal life years plus 55 maternal), which were then multiplied by a utility score. A completely healthy dyad was assigned a utility of 1, maternal death with a healthy child was assigned 0.03, and healthy mother but permanent brachial plexus injury to the child was assigned 0.6. These values were obtained from expert opinion.

The exact value judgements implied by this approach are difficult to disentangle. The combined utility values allow for maternal and fetal health outcomes to both be valued, and with varying weight relative to each other. However the justifications behind the individual values chosen were not presented. Dyad approaches potentially allow for interdependence between maternal and fetal outcomes, relaxing a strong assumption made by the additive approach that these outcomes are independent from one another.

The remaining nine studies reported maternal and fetal outcomes, but did not report how these were combined.

**Table 2:** Example calculations of the 4 methods for combining maternal and fetal outcomes. M, maternal; F, fetal

Method	Example Maternal outcome (M)	Example fetal outcome (F)	Method formula	Result	Assumptions	Number of studies
Additive	20 QALYs	25 QALYs	M + F	45	• Pregnant person and fetus are separate individuals • All QALYs are equal	31
Maternal disutility: maternal outcomes only	20 life years	Lifetime maternal disutility of 0.1	AM, where A is the maternal disutility attributable to a poor fetal outcome.	18 QALYs	• Only maternal outcomes are included • Maternal quality of life is impacted by fetal outcomes	5 4 further studies combined this with an additive approach
Dual time horizon	10 QALYs (up to 10 year time horizon)	25 QALYs (up to lifetime horizon)	M + F	35 QALYs	• Additive, but impact of treatment effect lasts longer for fetus than pregnant person	3
Dyad	55 life years	76 life years	B(M + F), where B is the utility value assigned to the combination of outcomes.	In the case of brachial plexus injury: 0.6 (55 + 76) = 78.6 QALYs	• Additive on life expectancy • Fetus and pregnant person remain a lifelong dyad, rather than 2 individuals, with a constant utility	1

## Discussion

### Summary of findings

There do not appear to be consistent approaches for valuing maternal and fetal outcomes within the QALY or DALY framework. The process of constructing a QALY in pregnancy presents a unique challenge: how are the outcomes for both the pregnant person and fetus to be balanced against both each other and against a broader cost-effectiveness threshold? The findings of this review outline how health economists up to this point have tackled this challenge in practice, and what assumptions they have made in the process.

#### Are both maternal and fetal outcomes included?

The strongest assumption identified in this review is that interventions can be considered to affect only one member of the pregnancy dyad. More than half of studies (72 out of 127) chose to exclude either the fetus or pregnant person from the analysis. Fifty-nine papers excluded maternal outcomes. Whether this is justifiable depends on two considerations: first, whether the clinical evidence supports the assumption that maternal outcomes are unaffected by the intervention, and second, whether a change in fetal outcomes has an indirect effect on maternal HRQOL. This indirect effect is captured in nine studies that included a maternal disutility relating to fetal outcomes.

Only two studies gave a justification for excluding maternal outcomes, and two justified excluding fetal ones. Two main reasons were given for the exclusion: lack of evidence for an effect, and lack of clinical mechanism for an effect (for example, excluding maternal health outcomes when screening for fetal genetic disorders). For the vast majority of papers, a justification was not provided. However, in a number of cases, the exclusion of a set of outcomes does not seem plausible. This is particularly noticeable in the case of evaluations to screen and treat maternal infections. For example, 13 studies evaluated interventions for treating maternal infections such as HIV and syphilis, but reported that the sole target for the intervention was the fetus, and only included fetal outcomes in the analysis.

#### How are fetal QALYs constructed?

The majority of studies (*n* = 113) included fetal outcomes in their analysis. Most commonly, this involved extrapolating from the point of delivery using life expectancy data. Health effects from interventions therefore captured morbidity (through utility decrements lasting all or some portion of that life expectancy), deaths or losses during pregnancy and shortly after, and reduced life expectancy. Under this approach, the QALY loss attributable to a stillbirth is the discounted QALY value of an entire lifetime – quite literally the highest possible value. Many studies also assumed this life was lived in perfect health, with a utility of 1.

The assumptions implied by this approach are:

1. That the fetus is a wholly separate individual with equivalent personhood to any other individual receiving care,
2. That avoiding a fetal death is of more value than any other patient’s death, simply by virtue of the remaining life expectancy,
3. That the QALYs gained by averting fetal loss during pregnancy are at least as great as averting a neonatal death.

These assumptions are worthy of interrogation. Maternal and fetal outcomes are occasionally affected by interventions in opposing directions, and in these cases prioritising fetal outcomes over maternal ones may not be an appropriate choice [2]. An example of this is timing of delivery in hypertensive pregnancy, where an early delivery reduces the risk of poor maternal outcomes but increases risk to the fetus [8]. More broadly, assigning the highest possible QALY value prioritises fetal loss over all other health outcomes. More evidence is needed to ascertain whether this is an appropriate valuation according to societal preferences. If so this also needs to reconciled other societal priorities such as reproductive autonomy.

Six studies in this review sought to address these three assumptions by making use of the maternal disutility approach to valuing fetal outcomes. Under this model, the fetus is not treated as an individual, but rather as a component of maternal health outcomes; essentially a disutility of bereavement. This avoids the question of personhood, but conversely places an extremely small value on fetal outcomes. Indeed, treating fetal outcomes solely as a product of maternal ones assumes that there is no familial or societal value placed on new births, outside the personal desires of the pregnant person.

### How are maternal and fetal outcomes combined

Four approaches for combining maternal and fetal outcomes were identified. The additive approach assigns equal value to the fetus as an independent patient, while the maternal disutility approach treats fetal outcomes as a component of maternal quality of life. The dyad method is essentially a modified additive approach, with quality of life assigned to the entire dyad. Finally the dual time horizon approach assumes that the health effects of an intervention last for a shorter period for at least one member of the dyad, but otherwise follows the additive approach.

One approach not captured in the published papers is that of weighting fetal QALYs to be explicitly lower than maternal QALYs. Such an approach was used in at least one NICE guideline, where the QALY value attributable to fetal loss was set at 10 QALYs, based on the expert opinion of the committee [27].

### Implications for future research

The findings from this review outline three key issues present in current CUAs of pregnancy: poor standards of reporting; variability in the valuation of QALYs and DALYs for the same clinical outcome, such as miscarriage or stillbirth; and inconsistency in the inclusion of maternal and fetal outcomes and how these are combined.

The first issue requires that authors adhere to reporting standards outlined in good practice guidelines such as CHEERS [28], and report methods in sufficient detail to allow replication. The second could similarly be ameliorated by following best practice for the conduct of economic evaluations, namely including all relevant outcomes and applying relevant age-adjustment to long-term quality of life outcomes [29].

The third issue requires considering whether current approaches to valuing fetal outcomes are consistent with maternal, familial, clinical and societal preferences. A substantial stated preference literature in pregnancy exists [30, 31]. However, we found that only three studies made use of stated preference data in their analysis [25, 32, 33]. All used them to assign utility values to fetal outcomes, rather than to validate or moderate how fetal outcomes should be valued relative to other outcomes. Given the variation observed in the QALY loss attributed to fetal loss in particular, a theoretical framework that addresses the value of fetal outcomes within the context of health system resource allocation is needed. Such a framework would enable the evaluation of existing methods, as well as any new methods that are developed.

In addition to the stated preference literature, there is also a growing literature on the concept of spillover effects that may be valuable. Health spillovers refer to the indirect health consequences that accrue to family and informal carers [34]. Spillovers differ from pregnancy in several important ways, most notably in the independence of the individuals affected, but as a field it also faces ethical challenges where improving the health of one individual reduces the health outcomes of another [35].

In the absence of standardized methods, it is crucial that authors report the assumptions they are currently making in the comparative valuation of maternal and fetal health outcomes. We have identified a variety of approaches to this valuation, but the underlying assumptions of these approaches were not made explicit, which inhibits critical appraisal and further development.

### Limitations

This review was subject to a number of limitations. In particular, a large number of studies were identified due to the broad inclusion criteria. This limited the depth of data extraction possible. Likewise the scale of the search strategy meant that it was not possible to complete a full update of the searches before submission, with the resources available to the project. However, our study focusses on the methodology and theoretical approaches used to combine maternal and fetal outcomes and we are not aware of any studies using different approaches that have been published since our literature searches were conducted in December 2019. To verify this, we conducted forward citation tracking on the studies that reported novel methods to see if any studies had further developed these approaches, which did not find any relevant studies. While cost-utility analyses in this field have been published since our search date, we have not encountered any methodological studies focussed on developing this area.

Data relating to health valuation was not always clearly presented, and this may have reduced the accuracy of the findings. Assumptions were often implicit rather than explicit, which limits reproducibility.

## Conclusion

Pregnancy presents unique challenges to cost-effectiveness methods. What is the value of a baby being born in good health? Should all economic evaluations in pregnancy consider maternal health, or can that be excluded in certain circumstances? These questions need to be answered in order to ascribe values to pregnancy outcomes within economic evaluation. In their absence, strong assumptions have been made that may not be justifiable.

## Electronic supplementary material

Below is the link to the electronic supplementary material.

Supplementary Material 1

## Data Availability

The full list of included papers and the data extraction table generated during the current study is available in the appendix.

## References

[1] Abel L, Quaife M. A pregnant pause: rethinking economic evaluation in contraception and pregnancy. Value Heal. 2022;25:32–5. 10.1016/j.jval.2021.07.009 35031097

[2] Premkumar A, Gates E. Rethinking the Bioethics of pregnancy: time for a New Perspective? Obstet Gynecol. 2016;128:396–9. 27400011 10.1097/AOG.0000000000001509

[3] Kelley M. Counting stillbirths: women’s health and reproductive rights. Lancet [Internet]. 2011 [cited 2019 Mar 25];377:1636–7. http://www.ncbi.nlm.nih.gov/pubmed/21496905 10.1016/S0140-6736(11)60279-1 21496905

[4] Harris LH. Rethinking maternal-fetal conflict: gender and equality in perinatal ethics. Obstet Gynecol [Internet]. 2000 [cited 2019 Mar 25];96:786–91. http://www.ncbi.nlm.nih.gov/pubmed/11042319 10.1016/s0029-7844(00)01021-8 11042319

[5] Jamison DT, Shahid-Salles SA, Jamison J, Lawn JE, Zupan J. Incorporating deaths near the Time of Birth into estimates of the global burden of Disease. In: Lopez A, Mathers C, Ezzati M, editors. Glob Burd Dis Risk factors. New York: Oxford University Press; 2006. 21250371

[6] Duhig KE, Seed PT, Myers JE, Bahl R, Bambridge G, Barnfield S, et al. Placental growth factor testing for suspected pre-eclampsia: a cost-effectiveness analysis. BJOG Int J Obstet Gynaecol. 2019;126:1390–8. 10.1111/1471-0528.15855 PMC6771855 31240854

[7] Russell J, Greenhalgh T, Lewis H, Mackenzie I, Maskrey N, Montgomery J et al. Addressing the ‘postcode lottery’ in local resource allocation decisions: a framework for clinical commissioning groups. J R Soc Med [Internet]. 2013 [cited 2022 Apr 29];106:120. http://pmc/articles/PMC3618168/10.1177/0141076813479192 PMC3618168 23564895

[8] Frampton GK, Jones J, Rose M, Payne L. Placental growth factor (alone or in combination with soluble fms-like tyrosine kinase 1) as an aid to the assessment of women with suspected pre-eclampsia: systematic review and economic analysis. Chapter 5: Economic Analysis. Health Technol Assess (Rockv) [Internet]. 2016 [cited 2019 Nov 4];20:1–160. https://www.journalslibrary.nihr.ac.uk/hta/hta20870/10.3310/hta20870 PMC5165281 27918253

[9] Goldhaber-Fiebert JD, Brandeau ML. Evaluating Cost-effectiveness of Interventions That Affect Fertility and Childbearing. Med Decis Mak [Internet]. 2015 [cited 2017 Sep 20];35:818–46. http://www.ncbi.nlm.nih.gov/pubmed/25926281 10.1177/0272989X15583845 PMC4418217 25926281

[10] Simon J, Petrou S, Gray A. The valuation of prenatal life in economic evaluations of perinatal interventions. Health Econ [Internet]. 2009 [cited 2017 Jul 19];18:487–94. http://www.ncbi.nlm.nih.gov/pubmed/18615854 10.1002/hec.1375 18615854

[11] Abel L. How have outcomes been reported in cost-effectiveness analyses during pregnancy? [Internet]. PROSPERO. 2019. p. CRD42020157325. https://www.crd.york.ac.uk/prospero/display_record.php?ID=CRD42020157325

[12] Ungar WJ, Santos MT. The Pediatric Economic Database Evaluation (PEDE) Project. Med Care [Internet]. 2003 [cited 2018 Sep 17];41:1142–52. http://www.ncbi.nlm.nih.gov/pubmed/14515110 10.1097/01.MLR.0000088451.56688.65 14515110

[13] Research Institute at The Hospital for Sick Children. PEDE Database [Internet]. 2016 [cited 2016 Feb 22]. http://pede.ccb.sickkids.ca/pede/database.jsp

[14] Ouzzani M, Hammady H, Fedorowicz Z, Elmagarmid A. Rayyan-a web and mobile app for systematic reviews. Syst Rev [Internet]. 2016 [cited 2020 Apr 20];5:210. http://www.ncbi.nlm.nih.gov/pubmed/27919275 10.1186/s13643-016-0384-4 PMC5139140 27919275

[15] Adam T, Lim SS, Mehta S, Bhutta ZA, Fogstad H, Mathai M et al. Cost effectiveness analysis of strategies for maternal and neonatal health in developing countries. BMJ. 2005. p. 1107. 10.1136/bmj.331.7525.1107 PMC1283271 16282407

[16] Faye S, Cico A, Gueye AB, Baruwa E, Johns B, Ndiop M et al. Scaling up malaria intervention packages in Senegal: Using cost effectiveness data for improving allocative efficiency and programmatic decision-making. Malar J [Internet]. 2018;17:159. http://www.malariajournal.com/home/10.1186/s12936-018-2305-6 PMC5894199 29636051

[17] Trevillion K, Ryan EG, Pickles A, Heslin M, Byford S, Nath S et al. An exploratory parallel-group randomised controlled trial of antenatal Guided Self-Help (plus usual care) versus usual care alone for pregnant women with depression: DAWN trial. J Affect Disord [Internet]. 2020;261:187–97. 10.1016/j.jad.2019.10.013 10.1016/j.jad.2019.10.013 31634678

[18] Mistry H, Gardiner HM. The cost-effectiveness of prenatal detection for congenital heart disease using telemedicine screening. J Telemed Telecare. 2013;19:190–6. 23576807 10.1258/jtt.2012.120418

[19] Wastlund D, Moraitis A, Thornton J, Sanders J, White I, Brocklehurst P et al. The cost-effectiveness of universal late‐pregnancy screening for macrosomia in nulliparous women: a decision‐analysis. BJOG An Int J Obstet Gynaecol [Internet]. 2019 [cited 2019 Aug 7];126:1471-0528.15809. https://onlinelibrary.wiley.com/doi/abs/10.1111/1471-0528.15809 10.1111/1471-0528.15809 PMC6771727 31066982

[20] Wang X, Guo G, Zheng J, Lu L. Cost-effectiveness of option B + in prevention of mother-to-child transmission of HIV in Yunnan Province, China. BMC Infect Dis [Internet]. 2019;19:517. https://www.ncbi.nlm.nih.gov/pmc/articles/PMC6560771/pdf/12879_2019_Article_3976.pdf 10.1186/s12879-019-3976-5 PMC6560771 31185927

[21] Tilden EL, Lee VR, Allen AJ, Griffin EE, Caughey AB. Cost-effectiveness analysis of latent versus active Labor Hospital Admission for medically Low-Risk, term women. Malden, Massachusetts: Wiley-Blackwell;: Birth; 2015. pp. 219–26. 10.1111/birt.12179 26095829

[22] Hersh AR, Megli CJ, Caughey AB. Repeat screening for syphilis in the third trimester of pregnancy: a cost-effectiveness analysis. Obstet Gynecol. 2018;132:699–707. 30095767 10.1097/AOG.0000000000002795

[23] Hersh AR, Skeith AE, Sargent JA, Caughey AB. Induction of labor at 39 weeks of gestation versus expectant management for low-risk nulliparous women: a cost-effectiveness analysis. Am J Obstet Gynecol [Internet]. 2019;220:590.e1-590.e10. 10.1016/j.ajog.2019.02.017 10.1016/j.ajog.2019.02.017 30768934

[24] Cipriano LE, Barth WH Jr., Zaric GS. The cost-effectiveness of targeted or universal screening for vasa praevia at 18–20 weeks of gestation in Ontario. BJOG Int J Obstet Gynaecol. 2010. pp. 1108–18. 10.1111/j.1471-0528.2010.02621.x 20560948

[25] Rowley PT, Loader S, Kaplan RM. Prenatal screening for cystic fibrosis carriers: an economic evaluation. Am J Hum Genet. 1998. pp. 1160–74. 10.1086/302042 PMC1377475 9758600

[26] Culligan PJ, Myers JA, Goldberg RP, Blackwell L, Gohmann SF, Abell TD. Elective cesarean section to prevent anal incontinence and brachial plexus injuries associated with macrosomia - a decision analysis. Int Urogynecol J. 2005;16:19–28. 10.1007/s00192-004-1203-3 15647962

[27] 4 Evidence and interpretation. | Routine antenatal anti-D prophylaxis for women who are rhesus D negative | Guidance | NICE [Internet]. [cited 2022 Aug 26]. https://www.nice.org.uk/guidance/ta156/chapter/4-Evidence-and-interpretation#cost-effectiveness

[28] Husereau D, Drummond M, Petrou S, Carswell C, Moher D, Greenberg D et al. Consolidated Health Economic Evaluation Reporting Standards (CHEERS) statement. BMJ [Internet]. 2013 [cited 2014 Dec 10];346:f1049. http://www.bmj.com.ezproxy.york.ac.uk/content/346/bmj.f1049.full 10.1136/bmj.f1049 23529982

[29] Caro JJ, Briggs AH, Siebert U, Kuntz KM, ISPOR-SMDM Modeling Good Research Practices Task Force. Modeling good research practices–overview: a report of the ISPOR-SMDM Modeling Good Research Practices Task Force–1. Value Health [Internet]. 2012 [cited 2017 Dec 9];15:796–803. http://www.ncbi.nlm.nih.gov/pubmed/22999128 10.1016/j.jval.2012.06.012 22999128

[30] Gärtner FR, De Bekker-Grob EW, Stiggelbout AM, Rijnders ME, Freeman LM, Middeldorp JM et al. Calculating Preference Weights for the Labor and Delivery Index: A Discrete Choice Experiment on Women’s Birth Experiences. Value Heal [Internet]. 2015;18:856–64. 10.1016/j.jval.2015.07.005 10.1016/j.jval.2015.07.005 26409614

[31] Ryan M, Ratcliffe J, Tucker J. Using willingness to pay to value alternative models of antenatal care. Soc Sci Med [Internet]. 1997 [cited 2018 Oct 17];44:371–80. https://www.sciencedirect.com/science/article/pii/S0277953696001542?via%3Dihub 10.1016/s0277-9536(96)00154-2 9004371

[32] Yieh L, McEvoy CT, Hoffman SW, Caughey AB, MacDonald KD, Dukhovny D. Cost effectiveness of vitamin c supplementation for pregnant smokers to improve offspring lung function at birth and reduce childhood wheeze/asthma. J Perinatol [Internet]. 2018;38:820–7. http://www.nature.com/jp/index.html 10.1038/s41372-018-0135-6 PMC6414472 29785060

[33] Van Bellinghen LA, Dimitroff A, Haberl M, Li X, Manton A, Moeremans K et al. Is adding maternal vaccination to prevent whooping cough cost-effective in Australia? Hum Vaccines Immunother [Internet]. 2018;14:2263–73. 10.1080/21645515.2018.1474315 10.1080/21645515.2018.1474315 PMC6183273 29771574

[34] Al-Janabi H, Van Exel J, Brouwer W, Coast J. A framework for including family health spillovers in economic evaluation. Med Decis Mak. 2016;36:176–86. 10.1177/0272989X15605094 PMC4708618 26377370

[35] Dixon P, Round J. Caring for Carers: Positive and Normative Challenges for Future Research on Carer Spillover Effects in Economic Evaluation. Value Heal [Internet]. 2019;22:549–54. 10.1016/j.jval.2018.10.010 10.1016/j.jval.2018.10.010 PMC6524130 31104733

